# Supplementary figures and images for: Assessing the effect of compounds from plantar foot sweat, nesting material, and urine on social behavior in male mice, Mus musculus
Source: PLoS One. 2022 Nov 2;17(11):e0276844. doi: 10.1371/journal.pone.0276844 (PMC9629637; doi:10.1371/journal.pone.0276844)

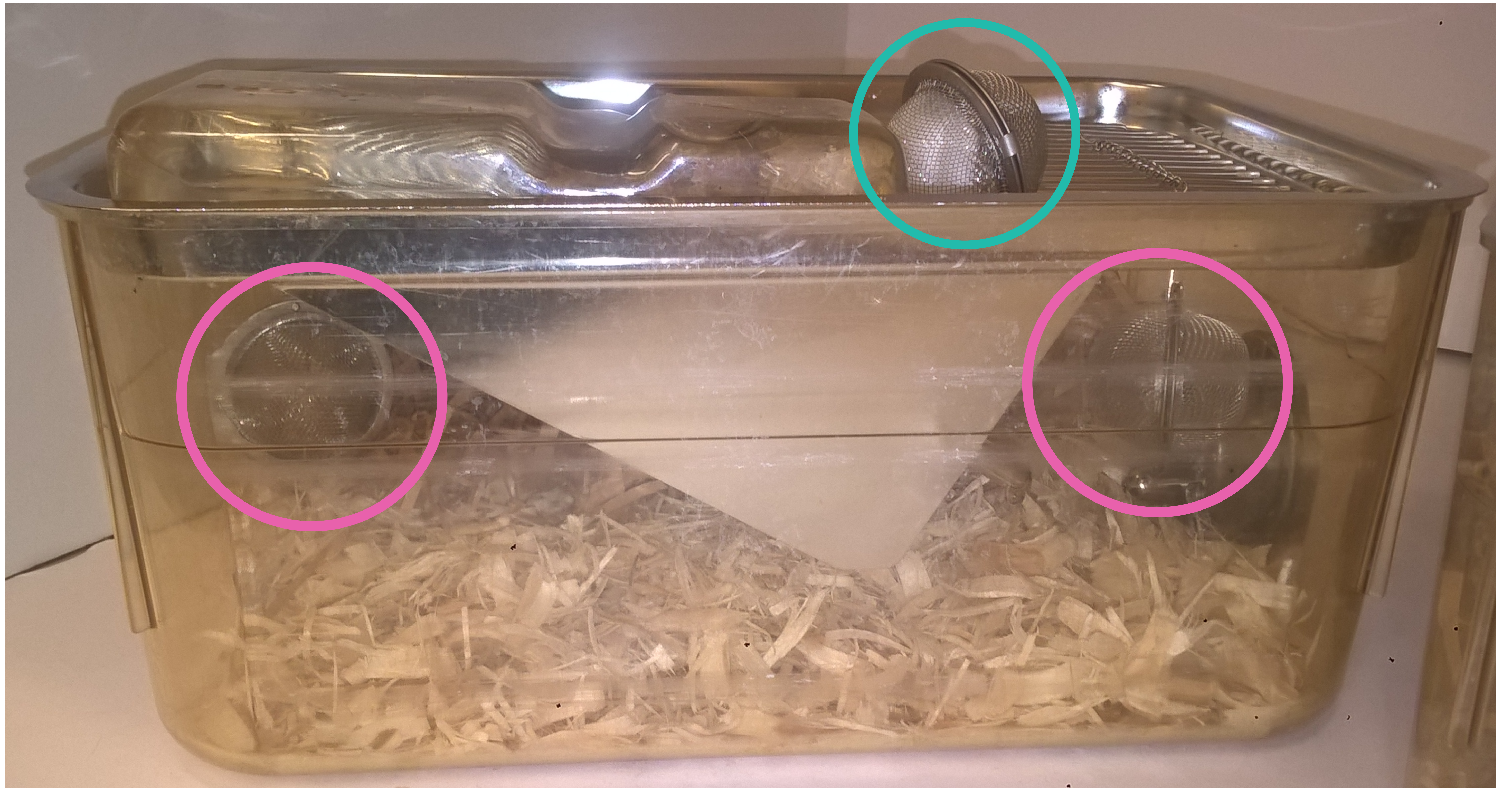

Supplement: S1 Fig — The teal circle indicates where the test compound solution was placed, and the pink circles indicate where the stir bars were placed for collection. (PDF) [file pone.0276844.s003.pdf]
