# Supplementary material for: Assessing the effect of compounds from plantar foot sweat, nesting material, and urine on social behavior in male mice, Mus musculus
Source: PLoS One. 2022 Nov 2;17(11):e0276844. doi: 10.1371/journal.pone.0276844 (PMC9629637; doi:10.1371/journal.pone.0276844)
Supplement: Attachment — Submitted filename: ReviewerComments20220811.docx [file pone.0276844.s005.docx]

Editor Comments

Dear Authors,

The reviewers have now commented on your manuscript. I suggest you to go through the comments and concerns raised by the reviewers. Most importantly, I personally found a lack of technical and scientific writing in the manuscript. Along with the reviewers comments, this could also be addressed/rectified.

# Reviewer #1

In this manuscript, Barabas and Gaskill et al. described the effects of pheromonal compounds from plantar foot sweat, nest material, and urine on social behavior in male mice. The aim of the study is fascinating and essential for the welfare of experimental animals; however, the reviewer thought that the manuscript is suited to publishing journals related to experimental animals. The reviewer recommends making appropriate figures and tables for publication; moreover, the main text might be fully edited for the convenience of the readers.

Thank you for this feedback. However, without specific recommendations, it is difficult to know what specific edits to make. We have made an attempt to make changes where possible.

Reviewer #2
The manuscript entitled “Assessing the effect of compounds from plantar foot sweat….. social behavior in male mice, Mus musculus” explores the VOCs correlate with behavior analysis. The flow of the manuscript is well written and provides useful information to the readers. The authors have carried out the follow-up work with well-structured data on the correlation of VOCs with mice behavior. The results are relevant with the objectives and presented well. The data are presented with appropriate photographs and tables. Further, the data were analyzed using suitable statistical approaches. However, the manuscript needs to address a few concerns which are listed below to improve the quality of manuscript.

Technical comments:
Abstract
4 previously identified compounds- include the compounds names in parentheses

We would like to include the compound names, but the journal’s abstract length limits make this difficult to include.
Respective compound solution? Technically not right.

This has been reworded.
Home cage social behavior …. Of the animals?
Yes, the journal’s abstract limit prevents us from adding additional text.

Volatile treatments? Do you mean the compounds?

Yes, this has been reworded.
B6 or c57BL/6N- Words should be used uniform throughout the text

This has been corrected.
Introduction:
The rationale for choosing 6-hydroxyt-6-mnethyl-3-heptanone should be clearly mentioned. There have been many compounds reported to have various behavioral effects in conspecifics.
We state in lines 76-79 that 6-hydroxy-6-methyl-3-heptanone was chosen because it previously correlated with allo-grooming in male mice.

It is unclear from the methods that whether the authors used one compound/one time, or mixed all the compounds in one cage. This should be clearly mentioned in the methods. And, if not mixed, reasons should be provided. Because, in natural conditions, all those compounds are mixed in the bedding material. Therefore, keeping this in mind, discussion should be revised at appropriate instances.

We state in lines 111-112 that there was only one compound per solution. It was also emphasized in lines 204-206. Further, this limitation is mentioned in the discussion lines 469-471.
Line no. 127-131. Appropriate figures could be included to enhance the readership

An additional figure of this setup is included in the supporting information. Please see S1 Fig.
Line no. 167- what is CIS4?

This has been clarified.
Treatments- should be included as a flow chart! This will improve the understanding of the readers. It should include (type of treatment, day of treatment, end of treatment, etc.) in a chart. The authors could utilize ARRIVE guidelines flow chart for this purpose.

We believe you are referring to the timeline, not flow chart, for point 9 of the ARRIVE guidelines? A timeline has been included as Fig 1.
Line no. 212- sat for ten minutes? Rewrite the sentences, as these lines are non-technical. Similar non-technical words exist in the manuscript. I suggest the authors to correct it.
This phrasing has been edited, but we cannot change non-technical terms if we are unsure which are considered non-technical. We have reviewed the manuscript to attempt to identify other lay terminology.

Table 2. include references (if applicable)

The website from which the descriptions were taken is provided in the table title. The website combines behavior definitions from several old publications, so to cite the original sources here would not be appropriate.
Fecal corticosterone metabolites
Is there any difference in the corticosterone conc. Between the first day and last day? Did the authors measure any differential data? Why it was done only on day 7?

Measures were not taken on the first day because it would likely be confounded with stress from shipping and or the first day of being combined in a new cage. Day 7 would provide us a better idea of the stress from potential fighting over the week. Fecal measures are generally considered more of a chronic than acute measurement.
Line no. 251-261- not necessary. But if the authors wish, they can trim these lines to a few and include.

This section has been condensed.
If the preputial gland data is presented, it should come under a separate subtitle (not under fecal corticosterone). Originally, line no 274 is the start point of fecal corticosterone analysis.

This data is not presented as it was solely used to determine which mice to sample. We stated that the mice with the highest and lowest values per cage were selected. The absolute measurement values did not play a role.
Line no. 281- The details of commercial kits (lot no., supplier, sensitivity, specificity) should be included.

A commercial kit was not used. The details of this assay are listed in the reference that has been provided.
Line no. 342- P<0.001 not p values<0.001

This has been corrected.
Figure 1 legends should be appropriately marked, because all the figures shows percent of active observations.

This has been added.
Line no. 455- delete “discussed above”

This was removed.
Line no. 464- if this rationale was not tested, it is better to remove this inference.

This is rationale as to why we did not see significant results (now line 475). As you mention above, these compounds are naturally found in a mixture in the environment and may work as a mixture. These lines address this previous comment. While we were not able to test this rationale, we think it is important to mention to the reader.
Line no. 479- This fact was not reported in the results section.

These results were reported in lines 350-351 and Fig 2C + 2F.

Line no. 510-514- discussion should be modified according to the presented results.

These lines have been removed.
Line no. 515-519- the real reason for this difference should be discussed and further implications of these results should be included.

We believe that the reasons for differences between strain fecal corticosterone metabolites is beyond the scope of this study. Strain is primarily included to control for potential variability that is inherent to strain not that we were wanting to determine why they have different stress levels.

General comments:
Reframe the sentences in line 47 ‘Only recently has it been’ rephrase the lines since it is not clear.

This sentence has been reworded.
In line 154, How the nesting material samples were set? Any rationale behind 0.58g?

We are not entirely sure what the reviewer’s question is about how samples were set. However, 0.58g was the amount that could fit within the sample tube.

Methods section contains various parameters like temperatures, degree symbols it is advised to recheck throughout the same. E.g., in line 163 the degree symbol is missing.

This symbol was added. We did not see any other instances of missing degree symbols.

The authors are mentioned in line 189, the animals are sacrificed and weight were calculated. If so, how the animals were sacrificed?

We refer in the previous sentence that “All mice were weighed at arrival and the end of the study.” However, to further clarify and point the reader to the correct section, we have added a sentence indicating that this information is present in the subsection “Wounding”.

The meaning of the abbreviations should be clearly defined at their mention in line no. 274.

The only abbreviation mentioned in that line is FCMs (now line 280), which was defined in line 261.

Please ensure that your manuscript meets PLOS One Journal style requirements to reference. In particular, the article title and that contain the species name should be in italics in line numbers 561, 565, 586. And also check line 611, 618 and so on.

We initially used the Vancouver style as recommended on the author guidelines. However, we found the PLOS ONE style and corrected the references.
Based on the above comments I recommended major revision of the manuscript before accepted for publication.
The manuscript should be checked regarding the technical information. Authors need to improve the quality of the paper by improving both scientific and technical writing.
